# Structural Analysis of Human Serum Albumin in Complex with the Fibrate Drug Gemfibrozil

**DOI:** 10.3390/ijms23031769

**Published:** 2022-02-04

**Authors:** Stefano Liberi, Sara Linciano, Giulia Moro, Luca De Toni, Laura Cendron, Alessandro Angelini

**Affiliations:** 1 Department of Biology, University of Padua, Viale G. Colombo 3, 35131 Padua, Italy; stefano.liberi@iusspavia.it; 2 Department of Molecular Sciences and Nanosystems, Ca’ Foscari University of Venice, Via Torino 155, 30172 Mestre, Italy; giulia.moro@unive.it (S.L.); sara.linciano@unive.it (G.M.); 3 Department of Medicine, Unit of Andrology and Reproductive Medicine, University of Padova, Via Giustiniani 2, 35128 Padova, Italy; luca.detoni@unipd.it; 4 European Centre for Living Technology (ECLT), Ca’ Bottacin, Dorsoduro 3911, Calle Crosera, 30123 Venice, Italy

**Keywords:** gemfibrozil, fibric acid, fibrate, Lopid, serum albumin, Sudlow’s site, hypolipidemic drug, hyperlipidaemia, hypertriglyceridemia, hypercholesterolemia

## Abstract

Gemfibrozil (GEM) is an orally administered lipid-regulating fibrate derivative drug sold under the brand name Lopid^®^, among others. Since its approval in the early 80s, GEM has been largely applied to treat hypertriglyceridemia and other disorders of lipid metabolism. Though generally well tolerated, GEM can alter the distribution and the free, active concentration of some co-administered drugs, leading to adverse effects. Most of them appear to be related to the ability of GEM to bind with high affinity human serum albumin (HSA), the major drug-carrier protein in blood plasma. Here, we report the crystal structure of HSA in complex with GEM. Two binding sites have been identified, namely Sudlow’s binding sites I (FA7) and II (FA3–FA4). A comparison of the crystal structure of HSA in complex with GEM with those of other previously described HSA–drug complexes enabled us to appreciate the analogies and differences in their respective binding modes. The elucidation of the molecular interaction between GEM and HSA might offer the basis for the development of novel GEM derivatives that can be safely and synergistically co-administered with other drugs, enabling augmented therapeutic efficacies.

## 1. Introduction

Gemfibrozil (5-(2,5-dimethylphenoxy)-2,2-dimethylpentanoic acid; GEM) is an FDA-approved fibric acid derivative (fibrate) [1,2]. Since its introduction in the clinic in the early 80s, GEM has been used as a lipid-lowering drug for the treatment of hypertriglyceridemia, especially type IV and V hyperlipidaemia [3,4]. GEM is also approved for decreasing the risk of developing hyperlipoproteinemia-related coronary heart disease (Type IIb), particularly in patients without a history of subsisting coronary heart disease symptoms [1]. Mechanistically, GEM activates the peroxisome proliferator-activated receptor-alpha (PPARα), changing the expression profile of genes involved in lipid metabolism. This process results in decreased levels of both serum triglycerides and serum low-density lipoproteins (LDL) as well as an increase in high-density lipoproteins (HDL) [5,6]. As high amounts of LDL have been reported to induce the process of atherosclerosis, lowering the ratio between LDL cholesterol to that of HDL cholesterol might reduce the risk of developing atherosclerosis-related diseases [7,8]. In addition to the regulation of dyslipidaemia, GEM have been proposed as an immunomodulatory, anti-inflammatory, and anti-migratory drug [9]. In this regard, GEM has also been used as an orphan drug to treat children with late neuronal ceroid lipofuscinoses and other lipid storage diseases [10,11].

Though effective in numerous therapies and generally well tolerated, the co-administration of GEM with other drugs has been frequently associated with adverse effects. For example, simultaneous usage of GEM with hydroxymethylglutaryl-coenzyme A (HMG-CoA) reductase inhibitors, such as statins, has been associated with increased risk of developing myopathy and rhabdomyolysis [12,13,14]. Combination therapy of GEM with the anticoagulant warfarin (Coumadin^®^ among others) has been linked to an increased anticoagulant effect of the latter, leading to severe hypoprothrombinaemia and bleeding [15,16,17]. Co-administration of GEM with enzalutamide (Xtandi^®^) increases the risk of seizures [18]. Severe side effects were also observed when GEM was administered in combination with the antiviral drug dasabuvir (Exviera^®^), the antidiabetic drug repaglinide (Prandin^®^ among others), and selexipag (Uptravi^®^), a drug used to treat pulmonary arterial hypertension [19,20,21,22]. A possible explanation of these adverse effects might be related to the rapid absorption and high binding affinity of GEM (>98% bound) to human serum albumin (HSA), the main protein carrier in our body that has the intrinsic capability to bind, transport, and distribute a large variety of endogenous and exogenous compounds, including a wide range of drugs. According to this hypothesis, the possible competition of GEM with other co-administered drugs that share mutual HSA-binding sites might alter the respective processes of distribution, causing major exposure to the free, active drug [23]. To avoid these adverse effects, an in-depth comprehension of the HSA-drug binding mode is required. In this frame, a better understanding of the binding mode of GEM with HSA and its interaction with other HSA-binding drugs is expected to have implications in the development of superior GEM derivatives that could be synergistically co-administered, ultimately becoming safer for use [24]. Though some insights on the binding mechanism of GEM to HSA have been collected, including the possible binding stoichiometry of two molecules of GEM to HSA [25,26,27,28], a high-resolution structural analysis of the interaction between GEM and HSA is still lacking.

In this work, we applied X-ray crystallography to unveil the molecular basis of GEM binding to HSA. Importantly, GEM showed two primary binding sites corresponding to the drug sites Sudlow’s I and II. The analogies and differences of GEM binding mode were emphasised by comparison with other HSA-binding drugs.

## 2. Results

### 2.1. Overview of the Crystal Structure of HSA in Complex with GEM Ligands

**Figure 1:**
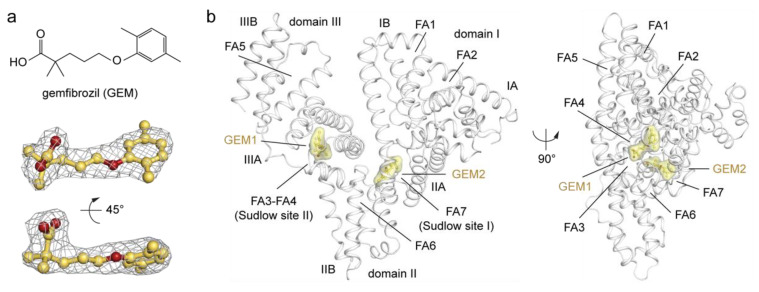
Structure of HSA in complex with GEM. (**a**) Chemical structure (top) and composite omit maps depicting the (*F*_o_−*F*_c_) electron density (bottom) of GEM1 ligand contoured at 2.5σ and shown in two orientations (90° rotation); (**b**) crystal structure of the HSA-GEM complex (white) shown in two orientations (90° rotation, PDB identification code: 7QFE). The structure of HSA is organised in homologues domains (I, II, and III), subdomains (A and B), fatty acids binding sites (FA), and Sudlow’s binding sites. The α-helices of HSA are represented by cartoon loops (white). Bound GEM1 and GEM2 ligands are shown in a ball-and-stick representation with a semi-transparent van der Waals surface (yellow) and coloured by atom type (GEM: carbon = yellow orange, oxygen = firebrick). The electron density of GEM ligands is shown as a grey mesh. The three-dimensional structure models were generated and rendered using Pymol [29].

To unveil the binding mode of HSA to GEM, we applied X-ray crystallography and determined the structure of the complex (Figure 1a,b). The best crystals diffracted to 2.2 Å maximum resolution, and the structure was solved by molecular replacement (Supplementary Table S1; PDB identification code: 7QFE). The polypeptide chain of HSA could be traced unambiguously from H3 to L583. Two binding sites are occupied by GEM and are located at the Sudlow’s sites I (FA7, subdomain IIA) and II (FA3-FA4, subdomain IIIA; Figure 1). Electron density maps and refined occupancies of the two sites revealed full saturation (100%) for FA4, while those for FA7 revealed slightly less occupation (74%). The remaining four HSA-binding sites (FA1, FA2, FA5, and FA6) are occupied by myristic acid (Myr) and/or other precipitating molecules used in crystallisation trials.

### 2.2. Molecular Binding Mode of GEM Ligands to HSA

**Figure 2:**
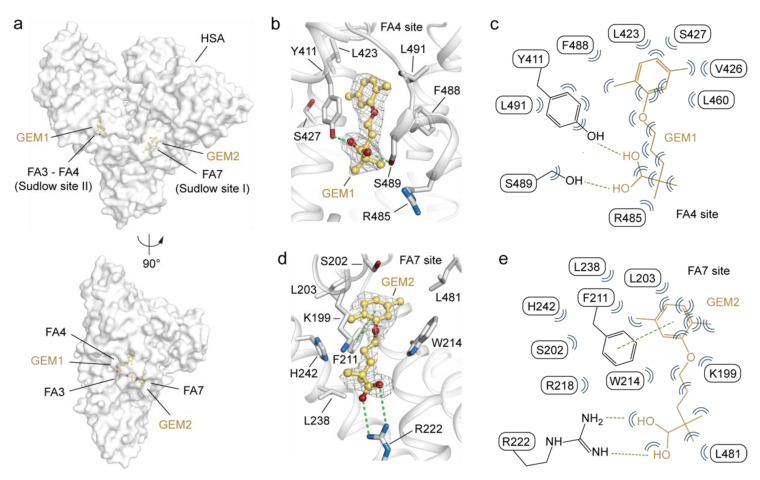
Details on the binding mode of GEM ligands to HSA. (**a**) Molecular surface representation of the overall HSA-GEM complex (white) shown in two orientations (90° rotation); (**b**) detailed view of GEM1 bound to FA4 in sub-domain IIIA; (**c**) schematic representation of molecular interactions between HSA residues and GEM1; (**d**) detailed view of GEM2 bound to FA7 in sub-domain IIA; (**e**) schematic representation of molecular interactions between HSA residues and GEM2. The α-helices of HSA are depicted as cartoon loops (white), and the selected labelled amino acid side chains are represented as sticks and coloured by atom type (carbon = white, oxygen = firebrick, and nitrogen = sky blue). Bound GEM molecules are depicted as ball-and-stick models coloured by atom type (carbon = yellow orange and oxygen = firebrick). The composite omit maps representing the (*F*_o_−*F*_c_) electron density of GEM ligands are contoured at 2.5σ and shown as a grey mesh. Hydrogen bonds, salt bridges, and π–π intermolecular interactions are shown as green dashed lines. Bent blue lines indicate residues of HSA in close contact with GEM ligands (distances shorter than 4.0 Å that are not hydrogen bonds). The three-dimensional structure models were generated and rendered using Pymol [29].

The electron density of the hydrophilic carboxylate head-group of GEM ligand bound to Sudlow’s site II (FA3-FA4), referred to as GEM1, could be univocally assigned while that of the hydrophobic 2,5-dimethylphenoxy tail is less clear (Figure 2a,b). The GEM1 molecule lies in the FA4 site roughly positioned at a right angle to the fatty acid Myr bound to FA3 (Figure 2b). Similar to fatty acids, the carboxylate moiety of GEM1 forms a hydrogen bond with the side chains of Y411 and S489 (Figure 2b,c and Supplementary Table S2). The rest of the molecule accommodates in the hydrophobic tunnel and establishes non-polar contacts with surrounding L423, V426, S427, L460. R485, F488, and L491 residues (Figure 2b,c and Supplementary Table S2). Contrarywise, the electron density of GEM ligand bound to Sudlow’s site I (FA7), indicated as GEM2, is less defined, allowing for a certain assignment of the position of only the 2,5-dimethylphenoxy moiety (Figure 2a,d). The carboxylate head-group of GEM2 forms a salt bridge with the side chain of R222 and a π–π interaction with the aromatic ring of F211 (Figure 2d,e and Supplementary Table S3). The rest of the molecule lodges in the hydrophobic pocket and engages in non-polar contacts with surrounding K199, S202, L203, F211, W214, R218, R222, L238, H242, and L481 residues (Figure 2d,e and Supplementary Table S3).

### 2.3. Structural Comparison of the Ligand Binding Modes of GEM and Other Known Drugs Binding HSA Sudlow’s Sites I and II

**Figure 3:**
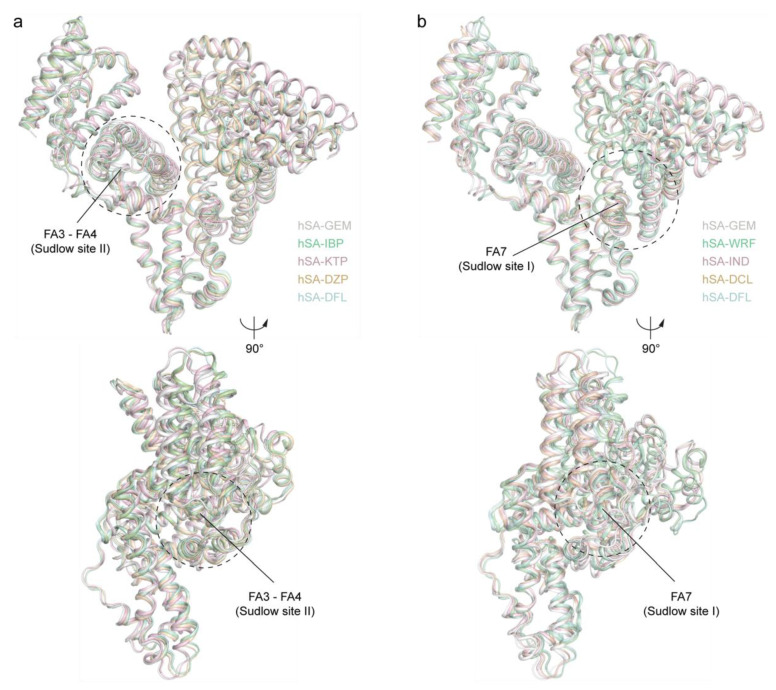
Structural comparison of different HSA-drug crystal structure complexes. (**a**) Superimposed HSA-GEM complex (white; PDB identification code: 7QFE) aligned with HSA-ibuprofen (pale green; PDB identification code: 2BXG), HSA-ketoprofen (light pink; PDB identification code: 7JWN), HSA-diazepam (wheat; PDB identification code: 2BXF), and HSA-diflunisal (pale cyan; PDB identification code: 2BXE) complexes shown in two orientations (90° rotation); (**b**) superimposed HSA-GEM complex (white; PDB identification code: 7QFE) aligned with HSA-warfarin (pale green; PDB identification code: 2BXD), HSA-indomethacin (light pink; PDB identification code: 2BXM), HSA-diclofenac (wheat; PDB identification code: 4Z69), and HSA-diflunisal (pale cyan; PDB identification code: 2BXE) complexes shown in two orientations (90° rotation). The α-helices of HSA are represented by cartoon loops and coloured. The three-dimensional structure models were generated and rendered using Pymol [29].

Next, we compared the binding mode of GEM ligands to HSA with that of eight different drugs known to bind HSA Sudlow’s sites I and II. If available, the comparison was performed using structures of HSA in complex with drugs recognised to have adverse effects when co-administered with GEM. If not, crystal structures of other significant HSA-binding drugs were considered. Overall, the superposition of multiple HSA-drug crystal structure complexes did not show any striking rearrangements of the main backbone with root mean square deviations of the Cα-atoms that never exceed 0.85 Å (Figure 3a,b) [30].

#### 2.3.1. Differences between GEM1 and Other Known Drugs Binding HSA Sudlow’s Site II (FA3–FA4)

**Figure 4:**
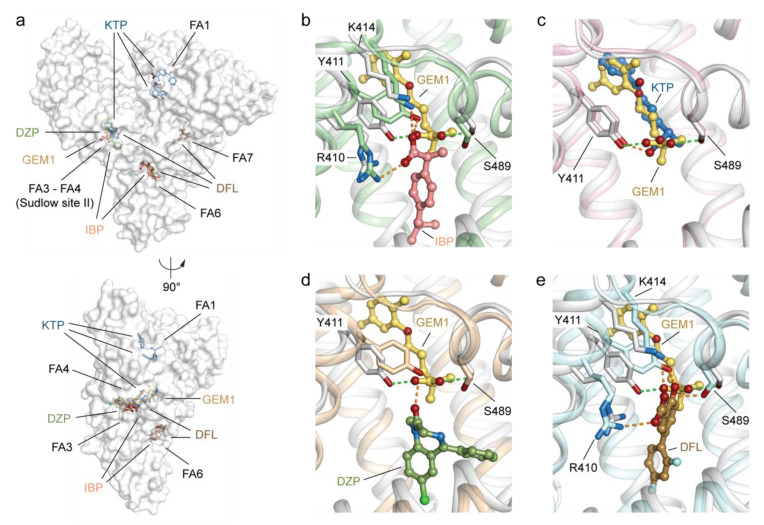
Structural comparison of the ligand binding mode of GEM1 with that of other known drugs binding HSA Sudlow’s site II (FA3-FA4) in sub-domain IIIA. (**a**) Molecular surface representation of the overall HSA-GEM complex (white; PDB identification code: 7QFE) aligned with HSA-ibuprofen (IBP; PDB identification code: 2BXG), HSA-ketoprofen (KTP; PDB identification code: 7JWN), HSA-diazepam (DZP; PDB identification code: 2BXF), and HSA-diflunisal (DFL; PDB identification code: 2BXE) complexes. The superimposed complexes are shown in two orientations (90° rotation). Detailed view of the superimposed GEM1 (yellow orange) to (**b**) IBP (salmon), (**c**) KTP (sky blue), (**d**) DZP (smudge), and (**e**) DFL (brown) molecules bound to Sudlow’s site II (FA4). The α-helices of HSA in complex with GEM1, IBP, KTP, DZP, and DFL are represented by cartoon loops and coloured in white, pale green, light pink, wheat, and pale cyan, respectively. The selected labelled amino acid side chains are represented as sticks and coloured by atom type (carbon = white for the HSA-GEM1 complex, pale green for the HSA-IBP complex, light pink for the HSA-KTP complex, wheat for the HSA-DZP complex, pale cyan for the HSA-DFL complex, firebrick for oxygen, and sky blue for nitrogen). Bound ligands are shown in a ball-and-stick representation and coloured by atom type (GEM1: carbon = yellow orange and oxygen = firebrick; IBP: carbon = salmon and oxygen = firebrick; KTP: carbon = sky blue, oxygen = firebrick, and nitrogen = sky blue; DZP: carbon = smudge, oxygen = firebrick, nitrogen = sky blue, and chlorine = light green; and DFL: carbon = brown, oxygen = firebrick, and fluorine = pale cyan). For visualisation, only the side chains of amino acids of HSA forming inter-molecular polar interactions below 4.0 Å are shown. Inter-molecular interactions are represented as dashed lines. Those established by GEM1 are coloured in light green, while those formed by IBP, KTP, DZP, and DFL are coloured in orange. The three-dimensional structure models were generated and rendered using Pymol [29].

The binding mode of GEM1 was compared with that of four previously described drugs known to be accommodated into Sudlow’s site II (FA3-FA4), namely ibuprofen (IBP; PDB identification code: 2BXG), ketoprofen (KTP; PDB identification code: 7JWN), diazepam (DZP; PDB identification code: 2BXF), and diflunisal (DFL; PDB identification code: 2BXE) [30,31,32,33]. In addition to Sudlow’s drug-binding site II, IBP binds FA6, DFL occupies FA6 and Sudlow’s site I (FA7), whereas two molecules of KTP are lodged in FA1 (Figure 4a). Notably, no significant differences are observed for the side chains of the amino acids of HSA interacting with the GEM1 ligand when compared with those of HSA in complex with IBP, KTP, DZP, and DFL. All the drugs examined here can establish hydrogen bonds with the hydroxyl group of Y411 located at the centre of FA4 (Figure 4b–e). Additional salt-bridge and hydrogen-bond interactions are mediated by HSA residues oriented toward a basic polar patch located at one end of the apolar pocket. These include R410 and K414, which establish interactions with IBP and DFL, while S489 can form contacts with GEM and DFL (Figure 4b–e). Major differences in the binding mode of GEM1 to Sudlow’s site II (FA3-FA4) with respect to IBP, KTP, DZP, and DFL can ascribed to either the presence or absence of fatty acids in the complexes. Site FA4 is contiguous to FA3, and together, they can bind up to two fatty acids, forming an approximately right-angle [34]. Previous studies have shown that fatty acids can provide steric hindrance and often induce conformational changes, thus affecting the binding mode of the nearby drug [35]. Indeed, the lack of fatty acids in FA3 allows IBU, DZP, and DFL to be lodged in the bottom of the FA4 pocket at the interface with FA3 in a region (L387-F403 and L430-L453) otherwise occupied by the carboxylate head of the fatty acid bound to FA4 and the methylene tail of the fatty acid bound to FA3 (Figure 4b–e). On the contrary, the presence of a fatty acid bound to FA3 in the HSA-GEM and HSA-KTP complexes prompt GEM and KTP to occupy the long, narrow hydrophobic tunnel of FA4 (V415-L430 and L457-L491) usually engaged by the methylene tails of FAs bound to this site (Supplementary Figure S1). This feature might be exploited in the design of novel GEM derivatives capable of binding the FA4 pocket without competing with other nearby bound drugs.

#### 2.3.2. Differences between GEM2 and Other Known Drugs Binding HSA Sudlow’s Site I (FA7)

**Figure 5:**
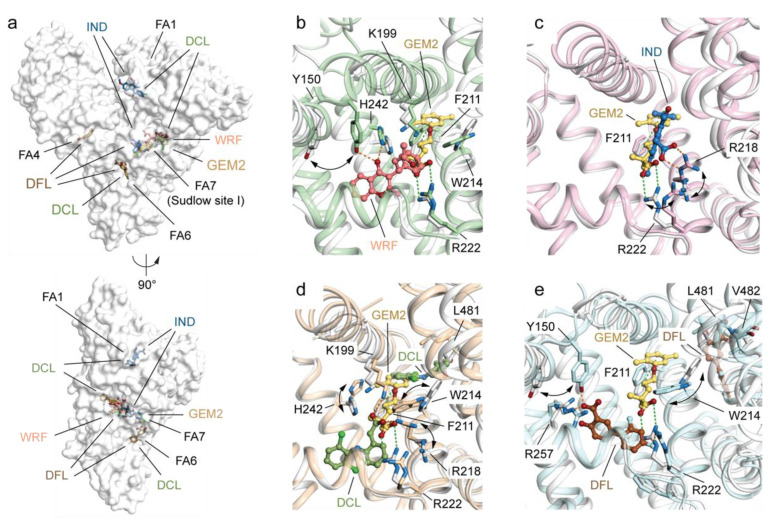
Structural comparison of the ligand binding mode of GEM2 with that of other known drugs binding HSA Sudlow’s site I (FA7) in sub-domain IIA. (**a**) Molecular surface representation of the overall HSA-GEM complex (white; PDB identification code: 7QFE) aligned with HSA-warfarin (WRF; PDB identification code: 2BXD), HSA-indomethacin (IND; PDB identification code: 2BXM), HSA-diclofenac (DCL; PDB identification code: 4Z69), and HSA-diflunisal (DFL; PDB identification code: 2BXE) complexes. The superimposed complexes are shown in two orientations (90° rotation). Detailed view of the superimposed GEM2 (yellow orange) to (**b**) WRF (salmon), (**c**) IND (sky blue), (**d**) DCL (smudge), (**e**) DFL (brown) molecules bound to Sudlow’s site II (FA4). The α-helices of HSA in complex with GEM2, WRF, IND, DCL, and DFL are represented by cartoon loops and coloured in white, pale green, light pink, wheat, and pale cyan, respectively. The selected labelled amino acid side chains are represented as sticks and coloured by atom type (carbon = white for the HSA-GEM2 complex, pale green for the HSA-WRF complex, light pink for the HSA-IND complex, wheat for the HSA-DCL complex, pale cyan for the HSA-DFL complex, firebrick for oxygen, and sky blue for nitrogen). Bound ligands are shown in a ball-and-stick representation and coloured by atom type (GEM2: carbon = yellow orange and oxygen = firebrick; WRF: carbon = salmon and oxygen = firebrick; IND: carbon = sky blue, oxygen = firebrick, and nitrogen = sky blue; DCL: carbon = smudge, oxygen = firebrick, nitrogen = sky blue, and chlorine = light green; and DFL: carbon = brown, oxygen = firebrick, and fluorine = pale cyan). For visualisation, only the side chains of amino acids of HSA forming inter-molecular polar interactions below 4.0 Å or presenting a notable difference are shown. Inter-molecular interactions are represented as dashed lines. Those established by GEM2 are coloured in light green while those formed by WRF, IND, DCL, and DFL are coloured in orange. The three-dimensional structure models were generated and rendered using Pymol [29].

Furthermore, the binding mode of GEM2 was compared with that of warfarin (WRF; PDB identification code: 2BXD), indomethacin (IND; PDB identification code: 2BXM), diclofenac (DCL; PDB identification code: 4Z69), and diflunisal (DFL; PDB identification code: 2BXE), four drugs known to be accommodated into Sudlow’s site I (FA7), among others (Figure 5a). Sudlow’s site I is larger than site II and includes numerous partially overlapping binding compartments, enabling lodging of multiple drugs concomitantly [30]. Indeed, Sudlow’s site I can host up to two molecules of DCL and DFL each, though located in distinct sub-chambers (Figure 5a). The binding mode of GEM2 resembles that of IND. Both drugs explore the same compartment of the pocket while WRF, DCL, and DFL access different hydrophobic sub-chambers located either at the right or left ends of the pocket (Figure 5b–e and Supplementary Figure S1). In addition to non-polar contacts, all drugs establish specific interactions with distinct polar residue clusters either at the bottom of the pocket (Y150, H242, and R257) or at its entrance (K195, K199, R218, and R222). WRF forms polar contacts with Y150 and H242, IND forms them with R218, DCL forms them with K199 and R218, while DFL engages in polar interactions with the side-chain of R222 and main-chains L418 and V481 (Figure 5b–e). Interestingly, the oxygens of the carboxylate moiety of GEM2 form salt-bridges with the amine of R222, an aspect that differentiates GEM2 from other drugs. Overall, Sudlow’s site I displays a comparatively greater side-chain movement associated with ligand binding than site II. Major variations were observed for the side chains of the Y150, W214, R218, and R222 residues (Figure 5b–e). In the case of the HSA-GEM2 and HSA-IND complexes, residue Y150 appears to be pushed away from the centre of the pocket while W214 undergoes a significant (~180°) rotation that allows the phenyl ring of both GEM2 and IND to access the rear righthand sub-chamber of the pocket, further maximising the π–π interaction between the aromatic rings (Figure 5b–e). Other meaningful shifts include turning of the side-chain R222 of HSA in complex with IND and the side-chain of R218 of HSA in complex with IND and DCL (Figure 5b–e).

## 3. Discussion

The evaluation of the efficacy and the safety profile of drugs co-administered with GEM requires a detailed analysis of the molecular interaction of GEM with HSA, the major drug-carrier protein in blood plasma. Toward this goal, here, we report the crystal structure of HSA in complex with GEM. Two binding sites have been identified, namely Sudlow’s binding sites I (FA7) and II (FA3–FA4). Sudlow’s site I, also known as the warfarin-azapropazone binding site, comprises a central zone from which three distinct compartments extend, while Sudlow’s site II, also known as the benzodiazepine binding site, is smaller and consists of a single narrow cleft [30]. Sudlow’s site I (FA7) usually accommodate dicarboxylic acids and/or bulky heterocycles carrying a central negative charge, whereas Sudlow’s site II (FA3–FA4) can discriminate ligands based on their size and stereoselectivity. Generally, aromatic carboxylic acids with a peripheric negative charge, that is distantly located from the hydrophobic centre, are lodged in the FA4 site [30]. Our data are in agreement with previous biochemical studies reporting that the majority of contraindicated drugs knowns to have adverse effects when co-administered with GEM appear to bind either or both Sudlow’s binding sites I and II [36,37,38,39,40]. Overall, the protein complex HSA-GEM was superimposed well with that of other well-known HSA-drug binding complexes examined here. A further detailed comparison of Sudlow’s binding sites I and II of HSA-GEM with that of other HSA-drugs complexes enabled us to appreciate analogies and differences in their respective binding modes. Major differences in the binding mode of GEM1 compared with IBP, KTP, DZP, and DFL can be ascribed to either the presence or absence of fatty acids in the complexes, a key aspect that cannot be ignored when analysing the interaction of HSA with drugs. Indeed, the binding of fatty acids to HSA can result in conformational changes and rotations of residues that ultimately affect the binding mode of the drug [35]. Differences and similarities were also observed in the binding mode of GEM2 to Sudlow’s site I, though no role is played by fatty acids in this case. The binding mode of different drugs to Sudlow’s site I appear to depend on the shapes and the distributions of few basic and polar residues located inside the hydrophobic pocket. The binding of GEM2 to Sudlow’s site I resembles that of IND while differ significantly from that of WRF, DCL, DFL, and other drugs known to bind to this site. The ability of GEM2 to explore patches of the pocket that are distinct from those recognised by most of the drug might be instrumental in the design of GEM derivatives with superior properties.

## 4. Materials and Methods

### 4.1. Protein and Chemicals

Recombinant human serum albumin (HSA, Albagen XL; UniProt ID: P02768) was purchased from Albumin Bioscience. The charcoal was purchased by Caesar & Loretz GmbH (Hilden, Germany). Sodium myristate (Myr) and gemfibrozil (GEM) were purchased from Cayman Chemical. All the reagents were of analytical grade. The stock solutions of GEM were prepared in pure DMSO. Myr was prepared in a solution of 50 mM sodium phosphate buffer (NaPi) and 100 mM NaCl, pH 7.4.

### 4.2. Protein Preparation and Purification

The defatted recombinant HSA was obtained by adsorption onto activated charcoal, as previously described [41]. Briefly, the water-washed charcoal (0.4 mg per mg of HSA) was initially dissolved in PBS pH 7.4 and the pH was further lowered to 3 using a 1 M HCl solution. The resulting suspension was incubated for at least 3 h under gentle shaking at 4 °C. The pH of the suspension was then adjusted to 7.4 using a 2 M NaOH solution and filtered using a 0.22 μm membrane filter. The protein aggregates and the disulfide-bridged dimers formed during this treatment were removed by size exclusion chromatography (SEC) using a HiLoad 16/600 Superdex 200 prep grade column (GE Healthcare, Chicago, IL, USA) connected to an ÄKTA pure 25 M system (GE Healthcare) equilibrated with 50 mM sodium phosphate buffer (NaPi) and 100 mM NaCl, pH 7.4. The fractions containing the monomeric HSA protein were pooled and further concentrated using 10,000 NMWL Amicon Ultra-15 ultrafiltration devices (Merck Life Science, Darmstadt, Germany) at 4000 g and 4 °C on a Heraeus Multifuge X1R centrifuge (Thermo Fisher Scientific, Waltham, MA, USA) to a final protein concentration of 25 mg mL^−1^ (375 μM). The protein concentration was determined using an Eppendorf BioSpectrometer^®^ (Eppendorf, Hamburg, Germany). Purified HSA protein was flash frozen in liquid nitrogen and stored at −80 °C. The monodisperse state of the concentrated HSA protein was confirmed by SEC using a Superdex 200 10/300 GL column (GE Healthcare, Chicago, IL, USA) connected to an ÄKTA pure 25 M system and equilibrated with 50 mM NaPi and 100 mM NaCl, pH 7.4. Purified HSA proteins were eluted as a single peak at elution volumes, which corresponds to an apparent molecular mass of about 66 kDa (monomer).

### 4.3. Crystallisation

Crystallisation trials of HSA in complex with GEM and/or sodium myristate (Myr) were carried out at 285 K in a SWISSCI MRC 96-well crystallisation plate (Hampton Research) using the sitting-drop vapor diffusion method and the Morpheus MD1-46 protein crystallisation screen kit (Molecular Dimensions Ltd., Sheffield, UK). Droplets of 0.8 μL volume (0.4 μL of protein complex and 0.4 μL of reservoir solution) were set up using an Oryx 8 crystallisation robot (Douglas Instruments Ltd., Hungerford, UK) and equilibrated against 75 μL reservoir solution. In all cases, the largest crystals were obtained by micro-seeding into drops that were allowed to equilibrate for 5-7 days. The best crystals of HSA incubated with a 10-fold molar excess of GEM and a 5-fold molar excess of Myr were obtained using the following precipitant agent: 50 mM bicine, 50 mM Trizma base, 30 mM diethylene glycol, 30 mM triethylene glycol (PGE), 30 mM tetraethylene glycol (PG4), 30 mM pentaethylene glycol, 12.5% *v*/*v* 2-methyl-2,4-pentanediol (MPD), 12.5% *w*/*v* PEG 1000, 12.5% *w*/*v* PEG 3350 pH 8.5, and 2.5% *v*/*v* DMSO. For X-ray data collection, the crystals were mounted on LithoLoops (Molecular Dimensions Ltd., Sheffield, UK) and flash frozen in liquid nitrogen.

### 4.4. X-ray Diffraction Data Collection and Processing

X-ray diffraction data of the complex were collected at ID30B beamline of the European Synchrotron Radiation Facility (ESRF, Grenoble, France). The best crystals were diffracted to 2.20 Å maximum resolution. The crystals belong to the C2 space group, with the following unit cell parameters: a = 184.90 Å, b = 38.64 Å, c = 96.21 Å, α = 90°, β = 104.58°, and γ = 90°. The asymmetric unit contains 1 molecule, corresponding to a Matthews coefficient of 2.56 Å^3^/Da and a solvent content of 51.93% of the crystal volume. The frames were indexed and integrated with software XIA2, merged, and scaled with AIMLESS (CCP4i2 crystallographic package) [42].

### 4.5. Structure Determination and Model Refinement

The structure was solved by molecular replacement with software PHASER [43] using the model 7AAI as a template [38]. Refinement was carried on using REFMAC [44] and PHENIX [45]. Rebuilding and fitting of the GEM, Myr, and precipitant/buffer molecules (MPD, PG4, and PGE) was performed manually with graphic software COOT [46]. Since the first cycles of refinement, the electron density corresponding to the bound GEM ligands was clearly visible in the electron density map. The final model of the complex contains 4626 protein atoms, 36 GEM ligand atoms, 32 Myr ligand atoms, 68 water molecules, and 76 atoms of other molecules. The final crystallographic *R* factor is 0.22 (*R*_free_ 0.25). The geometrical parameters of model are as expected or better for this resolution. Intra-molecular and inter-molecular interactions were analysed by PROFUNC [47], LigPlot+ [48], and Pymol software [29]. The Protein Data Bank (PDB) identification code for the HSA-GEM complex is 7QFE.

## 5. Conclusions

A detailed analysis of the molecular interaction of GEM with HSA is reported here. The crystallography data provide not only a reliable map of the locations and the binding modes of GEM to HSA but also offers the basis for the design of novel GEM derivatives that could be safely co-administered with other drugs to develop therapies with enhanced efficacy.

## Data Availability

Atomic coordinates and structure factors were deposited into the Protein Data Bank under the accession code 7QFE.

## Supplementary Materials

Additional supporting information are available online at https://www.mdpi.com/article/10.3390/ijms23031769/s1.
