# Supplementary material for: Structural Analysis of Human Serum Albumin in Complex with the Fibrate Drug Gemfibrozil
Source: Int J Mol Sci. 2022 Feb 4;23(3):1769. doi: 10.3390/ijms23031769 (PMC8836495; doi:10.3390/ijms23031769)
Supplement: Supplementary file 1 [file ijms-23-01769-s001.zip › ijms-1558272-supplementary.pdf]

Supplementary Materials

Structural analysis of human serum albumin in complex with the fibrate drug gemfibrozil

Stefano Liberi [1,†,‡], Sara Linciano [2,†], Giulia Moro [2], Luca De Toni [3], Laura Cendron [1,*] and Alessandro Angelini [2,4,*]

1 Department of Biology, University of Padua, Viale G. Colombo 3, 35131 Padua, Italy; stefano.liberi@iusspavia.it

2 Department of Molecular Sciences and Nanosystems, Ca' Foscari University of Venice, Via Torino 155, 30172 Mestre, Italy; giulia.moro@unive.it (S.L.); sara.linciano@unive.it (G.M.)

3 Department of Medicine, Unit of Andrology and Reproductive Medicine, University of Padova, Via Giustiniani 2, 35128 Padova, Italy; luca.detoni@unipd.it

4 European Centre for Living Technology (ECLT), Ca' Bottacin, Dorsoduro 3911, Calle Crosera, 30123 Venice, Italy

* Correspondence: laura.cendron@unipd.it (L.C.); alessandro.angelini@unive.it (A.A.)

† The authors contributed equally to this work.

‡ Present address: The Armenise-Harvard Laboratory of Structural Biology, Department of Biology and Biotechnology "L. Spallanzani", University of Pavia, Via Ferrata 9, 27100 Pavia, Italy.

Supplementary tables

**Data collection** *	HSA–GEM–Myr
Beamline	ID30B
Wavelength (Å)	0.9686
Space group	C2
**Cell parameters**	
a, b, c (Å); α, β, γ (°)	184.90, 38.64, 96.21; 90, 104.58, 90
Resolution (Å)	46.56 – 2.20 (2.27 – 2.20)
Unique observations	33831 (2911)
Multiplicity	3.2 (3.2)
R_{merge}	0.069 (0.561)
R_{pim}	0.058 (0.533)
$\langle I / \sigma(I) \rangle$	8.8 (1.7)
CC1/2	0.997 (0.753)
Completeness (%)	99.2 (98.6)
**Refinement**	
No. reflections (used for R_{free} calculation)	33826 (3419)
R_{work}/R_{free}	0.219/0.255
Number non-hydrogen atoms	4838
protein (chain A)	4626
ligands (GEM, Myr)	68
solvent	68
Others (MPD, PO4, PG4, PGE)	76

**Geometry**	
RMSD values	
bond lengths (Å)	0.008
bond angles (°)	1.398
Ramachandran plot (%)	
most favoured	94.82
additionally allowed	4.98
outliers	0.2
Rotamers outliers (%)	3.73
Average B-factor	56.0

Supplementary Table S1. Statistics on X-ray diffraction data collection and refinement. Frames were measured in 0.1° oscillation steps at 100 K. A single crystal was used to collect all diffraction data. The highest-resolution shell statistics are shown within brackets.

HSA—Sudlow's site II (FA3–FA4)	GEM1 ligand
**HSA atom/residue**	**atom, type of interaction, distance (Å)**
CB/Tyr411	C02 (NP, 3.77)
CG/Tyr411	C07 (NP, 3.65)
CD1/Tyr411	C06 (NP, 3.76)
CD1/Tyr411	C07 (NP, 3.85)
CD1/Tyr411	O08 (NP, 3.88)
CE1/Tyr411	O08 (NP,3.81)
CE1/Tyr411	C09 (NP, 3.52)
CE1/Tyr411	C10 (NP, 3.87)
CE1/Tyr411	O17 (NP, 3.40)
CZ/Tyr411	C09 (NP, 3.72)
CZ/Tyr411	O17 (NP, 3.33)
OH/Tyr411	O17 (HB, 2.45)
OH/Tyr411	C10 (NP, 3.89)
OH/Tyr411	C11 (NP, 3.46)
OH/Tyr411	C12 (NP, 3.83)
OH/Tyr411	C15 (NP, 3.47)
CD2/Leu423	C01 (NP, 3.90)
CD2/Leu423	C03 (NP, 3.77)
CB/Val426	C01 (NP, 3.49)
CG1/Val426	C01 (NP, 3.46)
OG/Ser427	C01 (NP, 3.87)
CD2/Leu460	C01 (NP, 3.89)
CD/Arg485	C13 (NP, 3.68)
CD1/Phe488	C18 (NP, 3.82)
CA/Ser489	O16 (NP, 3.25)
CB/Ser489	O16 (NP, 3.26)
OG/Ser489	O16 (HB, 2.60)
OG/Ser489	C13 (NP, 3.46)

OG/Ser489	C15 (NP, 3.70)
CD1/Leu491	C18 (NP, 3.45)

Supplementary Table S2. Inter-molecular interactions in the complexes between HSA and GEM1. Optimal inter-molecular hydrogen bonds (HB), polar interactions (PI), and non-polar interactions (NP) were defined using the web server PROFUNC [1] and LigPlot + [2].

HSA—Sudlow's site I (FA7)	GEM2 ligand
**HSA atom/residue**	**atom, type of interaction, distance (Å)**
CA/Lys199	C18 (NP, 3.89)
O/Lys199	C04 (NP, 3.55)
O/Lys199	C05 (NP, 3.86)
O/Lys199	C18 (NP, 3.56)
CB/Lys199	C18 (NP, 3.48)
CG/Lys199	C18 (NP, 3.68)
C/Ser202	C03 (NP, 3.76)
O/Ser202	C03 (NP, 3.79)
CB/Ser202	C03 (NP, 3.76)
N/Leu203	C03 (NP, 3.84)
N/Leu203	C04 (NP, 3.55)
CA/Leu203	C04 (NP, 3.79)
CD2/Phe211	C03 (NP, 3.69)
CD2/Phe211	C04 (NP, 3.14)
CD2/Phe211	C05 (NP, 3.02)
CD2/Phe211	C06 (NP, 3.44)
CD2/Phe211	C09 (NP, 3.63)
CD2/Phe211	C18 (NP, 3.36)
CE2/Phe211	C05 (NP, 3.35)
CE2/Phe211	C06 (NP, 3.72)
CE2/Phe211	C09 (NP, 3.23)
CE2/Phe211	C10 (NP, 3.38)
CE2/Phe211	C18 (NP, 3.12)
CD1/Trp214	C09 (NP, 3.62)
CG/Arg218	O17 (NP, 3.55)
NH1/Arg222	O16 (PI, 3.88)
NH2/Arg222	O17 (PI, 4.2)

CD2/Leu238	C13 (NP, 3.23)
CE1/His242	C14 (NP, 3.63)
NE2/His242	C14 (NP, 3.14)
CD2/Leu481	C01 (NP, 3.50)

Supplementary Table S3. Inter-molecular interactions in the complexes between HSA and GEM2. Optimal inter-molecular hydrogen bonds (HB), polar interactions (PI), and non-polar interactions (NP) were defined using the web server PROFUNC [1] and LigPlot+ [2].

Supplementary figure

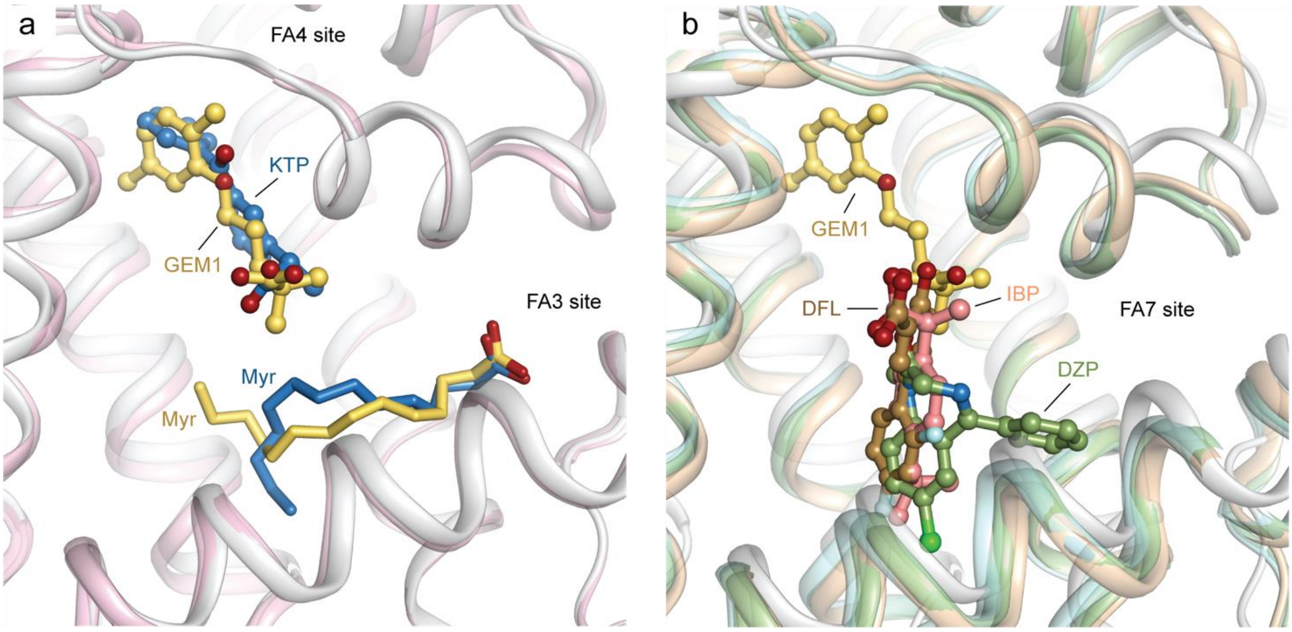

Supplementary Figure S1. **Comparison of the ligand occupancy at Sudlow's binding sites II (FA3–FA4) and I (FA7) in HSA–GEM and other ligands**. (**a**) Detailed view of the superimposed GEM1 (yellow orange) and ketoprofen (KTP, sky blue) bound to Sudlow's site II (FA3–FA4) crystallised in the presence of myristic acid (My). The α-helices of HSA in complex with GEM1 (PDB identification code: 7QFE) and KTP (PDB identification code: 7JWN) are represented by cartoon loops and coloured in white and light pink, respectively. Bound ligands are shown in a ball-and-stick representation and coloured by atom type (GEM1: carbon = yellow orange and oxygen = firebrick; KTP: carbon = sky blue, oxygen = firebrick, and nitrogen = sky blue; Myr co-crystallised in complex with GEM1: carbon = sky blue and oxygen = firebrick; Myr co-crystallised in complex with KTP: carbon = sky blue and oxygen = firebrick); (**b**) detailed view of the superimposed GEM1 (yellow orange), ibuprofen (IBP, salmon), diazepam (DZP, smudge), and diflunisal (DFL, brown) bound to Sudlow's site I (FA7) crystallised in the absence of myristic acid (My). The α-helices of HSA in complex with GEM1 (PDB identification code: 7QFE), IBP (PDB identification code: 2BXG), DZP (PDB identification code: 2BXF), and DFL (PDB identification code: 2BXE) are represented by cartoon loops and coloured in white, pale green, wheat, and pale cyan,

respectively. Bound ligands are shown in a ball-and-stick representation and coloured by atom type (GEM1: carbon = yellow orange and oxygen = firebrick; IBP: carbon = salmon and oxygen = firebrick; DZP: carbon = smudge, oxygen = firebrick, nitrogen = sky blue, and chlorine = light green; and DFL: carbon = brown, oxygen = firebrick, and fluorine = pale cyan). The three-dimensional structure models were generated and rendered using Pymol [3].

References

1. Laskowski, R.A.; Watson, J.D.; Thornton, J.M. ProFunc: a server for predicting protein function from 3D structure. *Nucleic Acids Res.* **2005**, *33*, W89–W93, doi:10.1093/nar/gki414.
2. Laskowski, R.A.; Swindells, M.B. LigPlot+: Multiple ligand-protein interaction diagrams for drug discovery. *J. Chem. Inf. Model.* **2011**, *51*, 2778–2786, doi:10.1021/ci200227u.
3. Janson, G.; Zhang, C.; Prado, M.G.; Paiardini, A. PyMod 2.0: improvements in protein sequence-structure analysis and homology modeling within PyMOL. *Bioinformatics* **2017**, *33*, 444–446, doi:10.1093/bioinformatics/btw638.
